# Cost and logistics implications of a nationwide survey of schistosomiasis and other intestinal helminthiases in Sudan: Key activities and cost components

**DOI:** 10.1371/journal.pone.0226586

**Published:** 2020-05-18

**Authors:** Mousab Siddig Elhag, Yan Jin, Mutamad Ahmad Amin, Hassan Ahmed Hassan Ahmed Ismail, Sung-Tae Hong, Hae In Jang, Youngah Doh, Seungman Cha

**Affiliations:** 1 Communicable and Non-Communicable Diseases Control Directorate, Federal Ministry of Health, Khartoum, Sudan; 2 Department of Microbiology, Dongguk University College of Medicine, Gyeongju, South Korea; 3 Research and Grants Unit, Ahfad University for Women, Omdurman, Khartoum, Sudan; 4 Department of Tropical Medicine and Parasitology, Seoul National University College of Medicine, Seoul, South Korea; 5 East Africa Department, Korea International Cooperation Agency, Sungnam, South Korea; 6 Department of Global Development and Entrepreneurship, Graduate School of Global Development and Entrepreneurship, Handong Global University, Pohang, South Korea; 7 Department of Disease Control, London School of Hygiene & Tropical Medicine, London, United Kingdom; Helen Keller International, UNITED KINGDOM

## Abstract

It is vital to share details of concrete experiences of conducting a nationwide disease survey. By doing so, the global health community could adapt previous experiences to expand geographic mapping programs, eventually contributing to the development of disease control and elimination strategies. A nationwide survey of schistosomiasis and intestinal helminthiases was conducted from December 2016 to March 2017 in Sudan. We aimed to describe details of the key activities and cost components required for the nationwide survey. We investigated which activities were necessary to prepare and conduct a nationwide survey of schistosomiasis and intestinal helminthiases, and the types and amounts of transportation, personnel, survey equipment, and consumables that were required. In addition, we estimated financial and economic costs from the perspectives of the donor and the Ministry of Health. Cash expenditures incurred to implement the survey were defined as financial costs. For economic costs, we considered the true value for society as a whole, and this category therefore accounted for the costs of all goods and services used for the project, including those that were not sold in the market and therefore had no market price (e.g., time spent by head teachers and teachers). We organized costs into capital and recurrent items. We ran one-way sensitivity and probabilistic analyses using Monte-Carlo methods with 10,000 draws to examine the robustness of the primary analysis results. A total of USD 1,465,902 and USD 1,516,238 was incurred for the financial and economic costs, respectively. The key cost drivers of the nationwide survey were personnel and transportation, for both financial and economic costs. Personnel and transportation accounted for around 64% and 18% of financial costs, respectively. If a government finds a way to mobilize existing government officials with no additional payments using the health system already in place, the cost of a nationwide survey could be remarkably reduced.

## Introduction

Investments in neglected tropical diseases (NTDs) mapping, and optimizing control and elimination have increased in the past decade in many parts of the world. For instance, the Coalition for Operational Research on Neglected Tropical Diseases (COR-NTD) was established in 2016 in collaboration between World Health Organization Regional Office for Africa, country partners, researchers and program implementers to provide coordination and generate synergies [1, 2]. Another example is the Global Trachoma Mapping Project (GTMP), which was kicked off in 2012 to estimate the prevalence of trachoma infection [3]. Still, the prevalence of NTDs across a nation remains unknown or patchy, or the data are outdated in many parts of the world [4, 5]. For instance, nationwide prevalence data of schistosomiasis and intestinal helminthiases are not available in some countries in sub-Saharan Africa [6]. Implementation of NTD control has been hampered by a lack of data on their geographical distribution and limited funds [7]. Operational experience with nationwide NTD surveys is limited, and this is particularly true for integrated surveys [8]. Therefore, it is vital to share the details of concrete experiences of conducting a nationwide survey, so that the global health community could adapt it to expand geographic mapping programs, eventually contributing to the development of control and elimination strategies with limited resources.

In recent years, several studies [8–13] have begun exploring the main drivers of NTD survey costs. The first study analyzed the cost of the integrated surveys for lymphatic filariasis, schistosomiasis, and soil transmitted helminthiasis (STH) infection conducted in South Sudan by Kolaczinski and colleagues [8]. They called on the global community to undertake similar studies to compare results across different settings. However, the survey in South Sudan was not conducted throughout the nation; instead, it was carried out only in one state. The other previous studies [9–13] were limited to analyzing the costs of trachoma or lymphatic filariasis surveys (e.g. the global trachoma mapping project), not surveys of schistosomiasis and intestinal helminthiases. We need to understand what activities are necessary to prepare and conduct a nationwide survey of schistosomiasis and intestinal helminthiases, and the types and amounts of transportation, personnel, survey equipment, and consumables that are required.

Sudan was one of the African countries with the largest unmapped areas of schistosomiasis and STHs, and it was necessary to update the prevalence data [14]. The World Health Organization conducted the first nationwide mapping of schistosomiasis in 1986, but it was a combination of patchwork surveys that were not concurrently carried out. We undertook a nationwide survey and mapping to describe the accurate geographical distribution of schistosomiasis and intestinal helminthiases, and the results were previously published [15]. The nationwide survey of schistosomiasis and other intestinal helminthiases, including STHs, was conducted from December 2016 through March 2017, in which 105,167 students participated from 1,772 schools in 183 districts of all 18 states of Sudan. We adjusted the World Health Organization guidelines and subdivided each district into 3 sub-districts based on proximity to water bodies (near: less than 1 km, moderate: 1–5 km, far: above 5 km from water bodies) to conduct on more targeted surveys, mapping, and mass drug administration (MDA). We defined an ecological zone as an area located within a similar distance from bodies of water in a district. In this previous study of the nationwide survey results, we showed that conducting MDA interventions at the ecological zone level was more cost-effective under all circumstances than doing so at the district level [15].

For instance, the cost for averting one disability-adjusted life year was US$586 when we assumed a one-time MDA intervention at the ecological zone level with 75% coverage and an 8% prevalence threshold, while it was US$708 at the district level with the same coverage and threshold [15]. We also noted that hyper-endemic areas were concentrated in states bordering South Sudan, Ethiopia, Central African Republic, and Eritrea, and we thus are appealing to the global community to develop a joint strategy in order to collaborate to disrupt disease transmission across borders. We call for program managers and district health officials along international borders to set up cross border meetings, share information from villages around the borders and put forward strategies for synchronized mass drug administration if necessary as early as possible. Herein, we aim to describe the details of the key activities and components required to prepare and undertake a nationwide survey of schistosomiasis and other intestinal helminthiases, and to analyze the costs of the nationwide survey. We compare the results with those of previously conducted surveys to obtain insights into the key cost drivers of a nationwide survey of schistosomiasis and other intestinal helminthiases and to contribute to the development of survey designs elsewhere. Sharing experiences of nationwide surveys is important for the global health community to expand global mapping activities [16]. To the best of our knowledge, this is the first study of its kind to examine the details of the activities, logistics, and costs of a nationwide survey of schistosomiasis and other intestinal helminthiases.

## Materials and methods

This nationwide survey was carried out under the umbrella of the SUKO project, which was the Sudan-Korea collaboration project of schistosomiasis and other intestinal helminthiases control supported by the Korea International Cooperation Agency (KOICA). The survey protocol has been described in detail elsewhere [17].

### Ethical statement

For this study, ethical approval was obtained from the Federal Ministry of Health, Sudan (FMOH/DGP/RD/TC/2016) and the Korea Association of Health Promotion (130750–20,164-HR-020). We obtained informed consent from the head teachers and assent from schoolchildren in written form. School teachers informed the parents about the survey details through students and checked for parental consent before launching the survey. Finally, the survey team could not collect stool and urine samples from students at five schools solely because the students’ parents did not consent for the students to bring stool and urine samples to the survey team. All of the parents who did not provide consent were parents of female students [15]. The five schools were all girls’ schools and belonged to one particular district in Red Sea State. We replaced them with other schools in the same district, which were boys’ schools or mixed schools. Since boys were found to be more likely to be infected with schistosomiasis, the exclusion of the five girls’ schools might have resulted in an overestimation of the prevalence in that particular district [15]. However, we believe that this replacement did not affect the validity of the nationwide prevalence since the number of students in the replaced schools only corresponds to 0.3% of the total sample size in the nationwide survey.

Data collection was undertaken using tablet PCs and the data were anonymized.

### Study area

Sudan is the third largest country in Africa, comprising 189 districts in 18 states. The estimated Sudanese population is 37.4 million in 2016, of whom 45.6% are children younger than 15 years and 3.9% are people aged above 59 years. The White Nile, the Blue Nile, and the River Nile flow through the country. Sixty-one percent of people have access to improved drinking water and 27% to improved sanitation. The study population was students aged 8–13 at 15,761 primary schools across the country.

### Sampling

We used two-stage random sampling for the nationwide survey. We applied probability-proportional-to-size sampling to select the schools. Twenty students from the second, fourth, and sixth grades were selected at each school. There were only one or two ecological zones in some districts. We used random sampling for schools and students to derive precise estimates of prevalence with a sufficient sample size. Finally, we surveyed 105,167 students from 1772 primary schools from 390 ecological zones in 183 districts of 18 states across Sudan. The nationwide survey was conducted from December 2016 to March 2017.

### Detection of schistosomes and other helminths

Stool and urine samples were processed within 24 hours. A training module was given to each state level team, which included color images of the various parasites expected. We used the Kato-Katz technique for the eggs of *Schistosoma mansoni* and other intestinal helminthiases using two smears [18]. We used centrifugation to examine *S*. *haematobium* eggs. Two slides were observed for each sample and a federal supervisor cross-checked the results of the examinations by state laboratory technicians to validate them. A total of 655 people were deployed for the survey. In total, 100,726 urine samples and 96,679 stool samples were collected among the 105,167 students interviewed.

### Coordination and supervision

The steering committee designated the Director of Community Health of the Federal Ministry of Health (FMOH) as the chief national coordinator. The 18 State Ministries of Health selected one state-level coordinator to facilitate the survey under the control of the chief national coordinator. We applied both internal and independent supervision. The FMOH assigned 36 federal-level supervisors to oversee the activities of state-level staff and to ensure the quality of the survey results, of whom 18 supervised laboratory examinations and the other 18 supervised school-based interviews and specimen collection. The 18 federal-level supervisors of laboratory examinations were all senior laboratory technicians, and they re-examined 10% of the slides examined by state-level laboratory staff for internal quality assurance. In addition, two senior technologists with doctoral degrees from the Blue Nile Institute and University of Gezira, and one parasitologist and molecular biologist (Professor, PhD) from Al Neelain University participated in the survey as independent supervisors. They rechecked 5% of the slides for external quality assurance. Overall, fewer than 0.01% of the re-examination results were found to be mismatched between the internal and external supervisors’ results and those of the state-level laboratory technicians.

### Training

One week’s training was conducted for 36 federal-level laboratory and field supervisors and 18 state coordinators prior to the survey. The training covered the details of the survey protocol, action plans, ethical issues, and communication and interviewing skills, and other topics. Shortly after this training, the federal-level supervisors and state coordinators trained state-level laboratory technicians and field data collectors in each state.

### Analysis

For cost analysis, we applied the same methods suggested by Kolaczinski and colleagues [8]. We estimated financial and economic costs from the perspectives of the donor and the Ministry of Health [19]. Cash expenditures incurred to implement the survey were defined as financial costs. For economic costs, we considered the true value for society as a whole, and this category therefore accounted for the costs of all goods and services used for the project, including those that were not sold in the market and therefore had no market price such as time spending of head teachers and teachers. We organized costs into capital and recurrent items [19]. The costs for survey equipment such as microscopes, centrifuges, and vehicles provided by KOICA or the Ministry of Health, Sudan were included as capital items, since these resources last longer than a year and can be re-used for future surveys. Recurrent items are the resources expected to be consumed or replaced within one year. The costs for daily allowances, survey consumables for diagnosis or specimen collection, and all the other various expenses that cannot be categorized as capital items, daily allowances, or survey consumables for diagnosis or specimen collection, such as insurance and accommodation for foreign experts, were included as recurrent items in the category of ‘others.’

We calculated the average financial daily cost using straight-line depreciation for capital items. The total number of survey days was considered for the capital costs. We took into account all the resources invested in the survey, including the components that were not paid for, such as vehicles and survey equipment provided by the Ministry of Health, Sudan and the opportunity costs of primary school teachers’ time spent on the survey. Capital items were discounted with a 3% discount rate over their lifespan. Daily economic costs for all capital items were calculated and multiplied by the number of days they were used for the survey. We used the same estimated lifespan for vehicles and survey equipment (4 and 2 years, respectively) that were used in South Sudan [8], since similar harsh climactic conditions are present in both Sudan and South Sudan. Data for costs were collected from the financial records of the project. We used an average exchange rate of 1 SDG = 0.15264 USD for the costs of survey activities between September 2016 and March 2017. We did not use shadow prices. To estimate the total value of goods or services, we multiplied their unit price by the total number of each item. For office overhead costs, we directly requested the implementing organization (Korea Association of Health Promotion) to share the actual amount with the study team [19]. We presented the costs by district, sub-district, schools, and individual levels to allow for comparison with other studies. For key activities, real-time weekly and monthly reports were documented with budget components. Details of a reference case scenario are outlined in S1 Table and S2 Table explains the cost categories used and the components included. We applied the same scenario that was used for costing in South Sudan [8].

We ran one-way sensitivity and probabilistic sensitivity analyses using Monte-Carlo methods to examine the robustness of the primary analysis results [19]. One-way sensitivity analysis was used to assess the impact that changes in a certain parameter would have on the costs. We used 10000 iterations for the probabilistic sensitivity analysis. Discount rates and the lifespan of vehicles and survey equipment were adjusted. For the one-way sensitivity analysis, we used similar values for each parameter to those used in the previous study [8] to make comparisons between the results from Sudan and South Sudan. We used a normal distribution of the life cycle ranging from 2 to 10 years for vehicles and from 1 to 5 years for equipment in the probabilistic sensitivity analyses.

In addition, we estimated the costs on the basis of the assumptions that the FMOH would not provide additional payments to government officials and that the FMOH would carry out the survey without any support from foreign experts. A cumulative probabilistic curve was examined for the cost of survey per ecological zone and district.

## Results

Table 1 shows the main activities conducted in the nationwide survey from its preparation to results-sharing. A steering committee was established to supervise the overall program, consisting of high-profile Sudanese government officials, the World Health Organization country office staff, and parasitologists or epidemiologists from both Sudan and Korea. The first committee meeting was held in October 2016 to reach a consensus on the study protocol. After the committee agreed on the protocol, a workshop was organized to train state-level laboratory technicians and potential state-level supervisors. Thirty-six laboratory technicians and 18 state government officials were invited from 18 states. An additional objective of this workshop was to examine the capability of state-level laboratory technicians in order to help the management team understand the required intensity and duration of training to be conducted prior to the survey. During the three-day workshop, they were trained and their capability was assessed through classroom-based lectures and field-based mock surveys. Overall, the state-level laboratory technicians’ capability was found to be satisfactory. Most of them were laboratory technicians working for the state-run central laboratory, and they had experience examining various parasites for their routine work. Therefore, they were appropriately equipped with the required skills to detect schistosomes and other intestinal helminths through microscopy and centrifugation. All personnel for the survey were recruited in December 2016, including 18 federal-level supervisors for laboratory examinations, 18 federal-level supervisors for field visit activities for specimen collection and questionnaire administration, 18 state-level supervisors; 218 state-level laboratory technicians, 244 state level field-based data collectors, 3 independent quality control supervisors, 3 federal-level operation team members, and 2 independent data management support team members. We hired a laboratory team comprising laboratory technicians and assistants, and deployed a field work team consisting of one interviewer and one specimen collector. The number of members of the laboratory teams ranged from 12 to 22, while the field work teams included 12 to 24 members by state. Details on the number of members of the laboratory and field work teams are presented in S3 Table.

**Table 1:** Main activities and financial costs.

Time	Day	Main activities	Purpose	Trainers/Facilitators	Participants	Cost (US$)	%
October 23~25, 2016	3	Workshop	Examine the capacity of laboratory technicians and state coordinators	Senior laboratory technicians of Blue Nile Institute (1 Professor, 1 PhD) Federal Ministry of Health (1 PhD, 1 MD) External specialists (2 Professors, 1 PhD) Project management staff (5)	State level laboratory technicians (36) State coordinators (18)	17,750	1.1%
October 23, 2016	1	Steering board meeting	Reach a consensus on the study protocol of a nationwide survey	Project management staff (5)	Board members (8)	1,448	0.1%
December 18~22, 2016	5	Workshop & Training	Orientation Introduce overall project Help to understand study methods Train data collection methods Train laboratory examination	Federal Ministry of Health (1 PhD, 1 MD) External specialists (2 Professors, 1 PhD, 1 MSc) Project management staff (5)	State supervisors (18) Federal Supervisors—Senior laboratory technicians (18)—Supervisors for specimen collection and household survey (18)	64,791	3.9%
December 31, 2016-January 10, 2017	1	Workshop	Introduce project; Obtain consent	State Coordinators	Head teacher from target schools (1811)	92,612	5.6%
December 31, 2016 ~ March 15, 2017	12–30	Nationwide survey	Carry out a nationwide survey on schistosomiasis and other helminthiases	State level laboratory technicians (218) State level data collectors (244) Federal level supervisors (36) Independent quality control supervisors (3) External specialists (1) Central level operation team (5) Independent data management support team (2)	105,167 students, 1772 schools in 390 ecological zones, 183 districts	1,465,902	88.4%
March 19 2017	1	Post-survey workshop	Share lessons learnt Collect suggestions and recommendations for a next round of nationwide survey Discuss details of a community-based survey	Federal Ministry of Health (1 PhD, 1 MD) External specialists (2 Professors, 1 PhD) Project management staff (5)	State coordinators (18) Federal Supervisors- Senior laboratory technicians (18)—Supervisors for specimen collection and household survey (18)	10,745	0.6%
March 20 2017	1	Steering board meeting	Share key findings Understand policy implications Discuss future plans	Project management staff (5)	Board members (8)	5,104	0.3%
Grand Total						1,658,352	100%

In December 2016, 36 federal-level supervisors and 18 state-level supervisors were trained for five days in Khartoum, the capital city. It was ensured that they understood the survey objectives, student sampling methods, and details of questionnaires. They practiced laboratory techniques at National Laboratory of Federal Ministry of Health, Sudan to examine samples for *S*. *haematobium*, *S*. *mansoni*, and nine other intestinal helminths, including STHs, using real parasite egg samples. A private consultant company was contracted to develop and install a program to facilitate the real-time management of data. The consultant trained the supervisors on how to enter and manage data with tablet PCs purchased by the project team. Prior to the survey, the head teachers of 1811 target primary schools were invited by the Ministry of Health at the state level. State-level supervisors helped them to understand the overall objectives and methods of the study, and obtained their consent with, and support for, the survey between late December 2016 and early January 2017. The state-level laboratory technicians and data collectors were trained by federal- and state-level supervisors. Thirty-six federal-level supervisors were deployed to each state for the entire period of the nationwide survey, during which they undertook daily monitoring and supervision, and reported real-time results to the central operation team.

The nationwide survey was conducted between December 31, 2016 and March 15, 2017. The starting and ending date varied depending on the state, and the duration of the survey in each location ranged from 12 days to 30 days. Immediately after the survey, in March 2017, federal- and state-level supervisors participated in a post-survey workshop conducted to collect and share lessons learned and experiences, and to make recommendations for future surveys. Finally, a steering committee meeting was held to share key findings, discuss future plans, and derive policy recommendations. A total of USD 1,658,352 (financial cost) was spent for the overall activities of the nationwide survey, including preparation, the post-survey workshop, and steering committee meeting, of which USD 1,465,902 (88%) was incurred by the actual survey.

**Fig 1:**
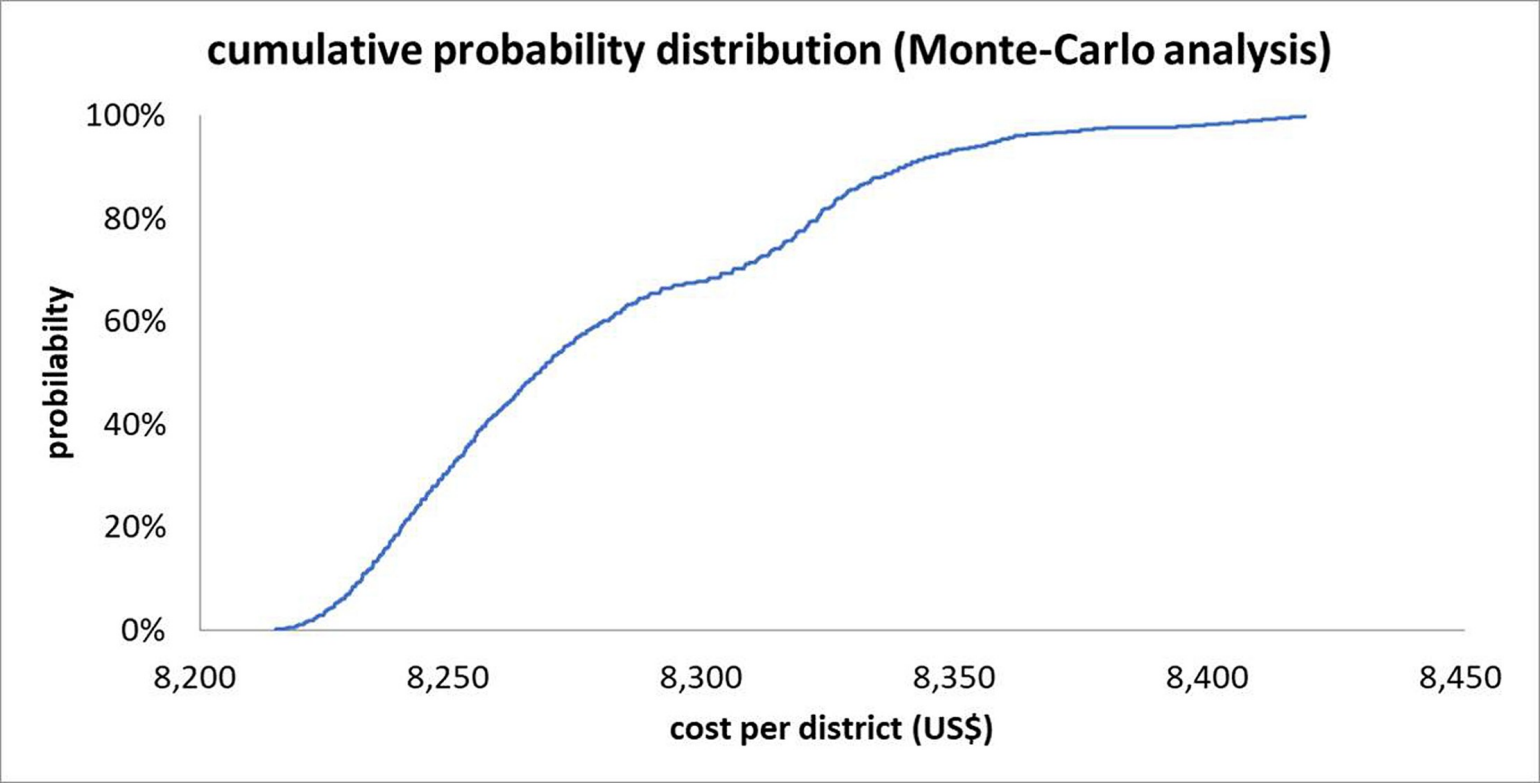
Cumulative probability distribution (Monte-Carlo probabilistic sensitivity analysis, 10,000 draws).

Table 2 shows the total financial and economic costs for the nationwide survey. A total of USD 1,465,902 and USD 1,516,238 was incurred for the financial and economic costs, respectively. The key cost drivers of the nationwide survey were personnel and transportation, for both financial and economic costs. Personnel and transportation accounted for around 64% and 18% of financial costs, respectively. Capital costs amounted to only 0.5% of the total financial costs and 1.4% of the total economic costs. There was a slight increase in capital economic costs, which mainly resulted from the opportunity cost of vehicles and survey equipment supported by the Ministry of Health, not paid for by the SUKO project. The fuel for those government vehicles was covered by the SUKO project and was already included in the financial costs. Seventeen government vehicles and 144 microscopes were provided by the Ministry of Health, Sudan with no charge for the SUKO project. Tables 3 and 4 present the details of financial and economic costs, respectively. For the survey, 422 vehicles × days and 3,464 microscopes × days were supported, which resulted in USD 10,886 and USD 2,877, respectively. In total, 5,316 school teachers were mobilized to facilitate specimen collections and questionnaire-based surveys; at one day per teacher, this was translated into USD 94,811 of economic costs. By evaluation unit, the economic cost was USD 8,285 per district or USD 3,888 per ecological zone. Based on the results of sensitivity analyses adjusting for the discount rate or lifespan of vehicles, the estimates did not change to a meaningful extent (Table 5, Fig 1).

**Table 2:** Financial and economic costs.

Items	Financial cost		Economic cost	
	US$	%	US$	%
**Capital costs**				
Survey equipment	7,256	0.5%	10,461	0.7%
Vehicles			10,886	0.7%
Sum	7,256	0.5%	21,347	1.4%
**Recurrent costs**				
Transportation (vehicle hiring)	260,479	17.8%	260,479	17.0%
Personnel (daily allowances)	942,272	64.3%	978,517	64.0%
Survey consumable	54,687	3.7%	54,687	3.6%
Others	170,104	11.6%	170,104	11.1%
Sum (recurrent cost)	1,427,542	97.4%	1,463,787	95.7%
Total (capital and recurrent cost)	1,434,798	97.9%	1,485,134	97.1%
Overhead	31,104	2.1%	31,104	2.0%
Grand	1,465,902	100.0%	1,516,238	99.1%
**Cost per unit**				
District	8,010		8,285	
Ecological zone	3,759		3,888	
School	827		856	
Person	14		14	

**Table 3:** Details of financial costs^a^.

Items		Unit	Quantity	Unit cost (US$)	Total cost (US$)
**Capital costs**					
Survey equipment	Microscope	EA	40	72	2,860
	Centrifuge	EA	57	35	1,968
	Refrigerator	EA	17	80	1,362
	Tablet PCs	EA	50	19	930
	Generator	EA	1	117	117
	Wifi Eggs	EA	2	9	18
	Sub-total			331	7,256
**Recurrent costs**					
Vehicle (Darfur)	Vehicle (rent, fuel & maintenance)	days	773	160	123,848
Vehicle (others)	Vehicle (rent, fuel & maintenance)	days	1,616	70	113,120
	MOH vehicle (fuel, per-diem)	days	422	56	23,511
	Sub-total				260,479
Personnel (daily allowances)	Federal-level operational team for managing real-time data	days	420	56	23,526
	Interviewers & sample collectors	days	7,332	33	245,421
	Laboratory technicians	days	7,585	45	343,196
	Laboratory assistants	days	2,523	34	85,008
	Cleaners	days	514	12	6,045
	State coordinators	days	514	50	25,521
	Federal supervisors	days	1,028	80	82,380
	Independent supervisors	days	21	120	2,516
	Foreign experts	days	222	152	33,849
	School teachers	days	5,316	18	94,811
	Sub-total	days			942,272
Survey consumable for diagnosis or specimen collection	Urine container	EA	200,000	0.027	5,400
	Stool container	EA	200,000	0.027	5,400
	Microscope slide tray	EA	400	7.000	2,800
	Slide	EA	600,000	0.024	14,460
	Cover slip	EA	200,000	0.003	680
	Pincette	EA	54	2.000	108
	Trans pipette (3 mL)	EA	300,000	0.029	8,700
	Trans pipette (1 mL)	EA	200,000	0.075	15,000
	Mess cylinder	EA	18	3.000	54
	Wood stick	EA	50,000	0.008	400
	Conical tube	EA	2,000	0.045	90
	Tube rack	EA	2,000	0.015	30
	Phenol	Bottle	18	18.750	338
	Glycerol	Bottle	9	25.000	225
	Malachite green oxalate	Bottle	4	10.000	40
	Plastic box	EA	36	1.870	67
	Mesh membrane	EA	100	6.250	625
	Mask	Box	54	5.000	270
	Sub-total				54,687
Others	T-shirts (interviewers and sample collectors)	EA	400	15.000	6,000
	Hat (interviewers and sample collectors)	EA	400	8.000	3,200
	Gloves (interviewers and sample collectors)	Box	90	3.130	282
	Supervision manual for federal-level supervisors	EA	36	20.000	720
	Poster (bench aids for diagnosis of intestinal parasites of WHO)	EA	20	10	200
	Questionnaire (printed version for contingency)	N/A	NA/	N/A	629
	Stationary (A4 sheets, pencils, notes for contingency)	N/A	N/A	N/A	595
	Delivery of survey consumables and equipment from Khartoum to each state	N/A	N/A	N/A	3,774
	Accommodation for federal-level supervisors	days	1,028	44	44,876
	Accommodation for foreign experts	days	222	80	17,760
	Food items for foreign experts	days	222	60	13,320
	Communication (air charge)	days	90	15	1,349
	Visas for foreign experts	frequency	6	79	472
	Insurance for foreign experts	frequency	6	32	189
	Air tickets for foreign experts	frequency	6	2,028	12,170
	Software program for real-time data managing system using table PCs	N/A	N/A	N/A	50,000
	Other miscellaneous petty cash (e.g. taxi fees, drinks for occasional meetings)	N/A	N/A	N/A	5,086
	Sub-total				170,104
Sub-total (recurrent cost)					1,427,542
Sub-total (capital + recurrent cost)					1,434,798
Overhead					31,104
Grand total					1,465,902

^**a**^the number of days for each item: S4–S7 Tables

**Table 4:** Details of economic costs.

	Items	Unit	Quantity	Unit cost (USD)	Total cost (USD)
**Capital costs (covered by KOICA funding)**					
Survey equipment	Microscopes	EA	40	75	2,990
	Centrifuges	EA	57	36	2,057
	Refrigerators	EA	17	84	1,424
	Tablet PCs	EA	50	19	973
	Generators	EA	1	122	122
	Wifi-eggs	EA	2	10	19
	Sub-total			346	7,584
**Capital costs (covered by the Ministry of Health, Sudan)**					
Vehicles		EA	17 vehicles, 422 days	25.8	10,886
Microscopes		EA	144 microscopes, 3,464 days	0.8	2,877
Sub-total					13,763
Total capital cost					21,347
**Recurrent costs (covered by KOICA funding**^**)**^^**a**^^ ^					1,427,542
**Personnel (covered by the Ministry of Education)**		days	5,316 teachers, 5,316 days	6.8	36,245
Sub-total (recurrent cost)					1,463,787
Sub-total (capital + recurrent cost)					1,485,134
Overhead					31,104
Grand total					1,516,238

^a^The detailed items and values of recurrent costs covered by KOICA are identical to those in Table 3.

**Table 5:** Sensitivity analysis to cost estimates.

Assumptions tested			District level	% Deviation from base case	Ecological zone level	% Deviation from base case
**One-way**	Base case		8,285		3,888	
	Discount rate	1%	8,282	-0.04%	3,886	-0.05%
		10%	8,303	0.22%	3,896	0.21%
	Vehicle lifespan	8 years	8,258	-0.33%	3,875	-0.33%
	Equipment lifespan	4 years	8,259	-0.31%	3,875	-0.33%
	**Special circumstances**					
	*No additional payments for government officials*		4,204	-49.3%	1,972	-49.3%
	*No foreign experts involved*		7,585	-8.4%	3,559	-8.5%

However, the sensitivity analysis showed that the costs could be reduced by almost 50% if the government could mobilize government officials for the survey without providing any additional payments. Similarly, the Ministry of Health could reduce the costs by about 8% if it could carry out the survey without any support from foreign experts.

The results of the Monte Carlo analysis with no assumption of special occasions such as ‘no additional payment to government officials’, or ‘no foreign experts’ with 10,000 trials show a range of all the cost metrics. Fig 1 presents the cumulative density functions of the costs at the district and sub-district levels. The fifth percentile of the costs was US$ 8,215 and USD 3,855 at the district and sub-district level, respectively, while the 95th percentile reached USD 8,357 and USD 3,921.

## Discussion

We provided details of the nationwide survey of schistosomiasis and other intestinal helminthiases in terms of the key activities and costs in Sudan, therefore when planning to undertake future nationwide NTD surveys, the global health community could adapt and refine the activities and cost elements of the survey described herein.

The main cost drivers were recurrent costs: transportation (particularly hiring vehicles) and personnel. Prior to the survey, we assessed the capabilities of state-level laboratory technicians and data collectors. We invited 36 laboratory examiners and 18 state coordinators, who were purposively selected by the State Ministry of Health. Based on the assessment, we realized that there was a sufficient number of laboratory technicians qualified for the survey. If there had not been enough laboratory technicians with the required skills to detect schistosomes and other intestinal helminths through microscopy and centrifugation, the project team would have organized a more intensive training for a longer duration with closer supervision. Alternatively, having fewer trained laboratory technicians would require that few teams move from one area to another; which would result in more time for the field survey. Either option would have increased costs.

Additionally, Sudan had state-level laboratory facilities, many of which had microscopes and centrifuges, although the quantities were not sufficient. In some exceptional cases, such as North Kordofan, we had to operate a mobile laboratory due to the remoteness of some schools, but in general, laboratory work was carried out at state-run central laboratories. If a nationwide survey is to be conducted in settings with no local laboratory, more costs will be incurred for the laboratory setting, including the cost for equipment.

When designing the study protocol, the project team planned to purchase seven vehicles (six for the state-level survey team and one for the central-level operation team). However, vehicle purchase was not possible during the survey period, and therefore vehicles had to be hired from local rental-car companies, which led to a substantial rise in recurrent costs. Although the budget allocated for purchasing seven vehicles was not minimal, if they had been purchased, those vehicles could have been used by the Federal and State Ministry of Health, Sudan for additional surveys and/or other purposes such as providing essential health services by the Federal and State Ministry of Health. For this survey, substantial costs were paid only for the one-time survey because we had to rely on rental cars, which should be avoided in future surveys.

The reference case suggested by Kolaczinski and colleagues [8] was applied for this study. The nationwide survey of schistosomiasis and other intestinal helminthiases in Sudan was much less expensive than the survey conducted in 2009 in South Sudan, its neighboring country. The average economic cost of the schistosomiasis, STH, and lymphatic filariasis survey in South Sudan was USD 40,206 or USD 9,573 per district (county) or sub-district (*payam*), respectively. In Sudan the average economic cost was USD 8,285 or USD 3,888 respectively. In Sudan, the average economic cost per school or per student was USD 856 or USD 14, respectively. The results of the Monte-Carlo analysis demonstrated that the costs varied little across a range of discount rates and lifespans of vehicles and survey equipment (5–95th percentile of cumulative distribution: US$ 8,215–8,357 at the district level and US$ 3,855–3,921 at the sub-district level). However, the one-way analysis results showed that the costs could be reduced substantially if the survey could be conducted by government officials with no additional payments and without any support from foreign experts (i.e., doing so would require only 43% of the cost incurred for the survey that we carried out). We appeal to high-profile officials in the Ministry of Health in many NTD-endemic countries to pay attention to the feasibility of conducting a nationwide survey with much lower costs by mobilizing government officials within the scope of their job description without additional payments.

Many cost-related studies about schistosomiasis have been conducted; however, the majority of them were cost-effectiveness analyses, in which the authors investigated the cost-effectiveness of preventive chemotherapy against schistosomiasis or other interventions such as snail control. For instance, Croce and colleagues assessed the cost-effectiveness of a 10-year schistosomiasis control program in Cambodia on an empirical basis [20]. Lo and colleagues ran a computer-based simulation to assess the cost-effectiveness of various control interventions against schistosomiasis and concluded that including snail control with MDA was the most cost-effective intervention [21]. However, cost implications of the required logistics could be different depending on the landscape or accessibility issues of a country. Other schistosomiasis-related cost studies include a study of the cost of illness of *S*. *mansoni* infection in Brazil, and a cost study of diverse methods of diagnosing schistosomiasis in resource-poor settings [22]. Although Knowles and colleagues presented the itemized costs of a school-based survey for S. *haematobium* and *S*. *mansoni* in Malawi, they did not assess economic costs, and the data used for cost estimation were drawn from a survey carried out only in some parts of Malawi, as the main purpose of their study was to investigate the cost-efficiency of preventive chemotherapy by a survey design [23]. Unlike previous studies, the main purpose of this study was to understand key drivers of costs for a nationwide schistosomiasis survey, and we analyzed economic costs to understand the true value of the costs for society by taking into account the costs of all goods and services used for the project (e.g., the opportunity cost of the time teachers spent supporting specimen collection). We hope that this study might provide insights into how to conduct a nationwide survey in the future in similar low-resource settings.

Still, caution is needed when interpreting the results of this study because our findings cannot be generalized to every other context with a high prevalence of schistosomiasis and other intestinal helminthiases, which is the major limitation of this study. In particular, Sudan has a number of highly skilled laboratory technicians, and NTDs are prioritized by both the Federal and State Ministry of Health [24, 25], making it feasible to mobilize a considerable number of capable workforce for conducting the survey. Another limitation of this study lies in the number of samples, which also relates to the generalizability of the study. Unlike the conventional methods recommended by the World Health Organization, which call for making MDA decisions at the district level [26], we divided each district into smaller areas according to the proximity to water bodies to reflect the focalized nature of schistosomiasis prevalence [27–30]. In addition, we sampled a sufficient number of students to derive precise estimates of prevalence at the state level [31]. This led to sampling far more students at the district level than would have been done using the conventional method (250 students from five schools per district using conventional methods vs. 900 students from 15 schools per district in this study). If this survey had applied the conventional methods, the average economic cost per district would have amounted to the current cost per ecological zone of this study (i.e., USD 3,888). Applying the current WHO protocol, the total cost of a nationwide survey across Sudan would have been US$ 734,832, corresponding to 49% of the actual survey cost.

Caution is needed when interpreting the cost results of this study. The nationwide survey was conducted at the school level. However, a nationwide survey can be done at the community level in countries with a low school enrollment rate. Out-of-school children may have a higher prevalence of schistosomiasis than school-going children because they are more likely to come into contact with polluted water for household chores or for their livelihood. For those who are planning to conduct community based surveys for schistosomiasis in this context, it should be noted that they are more expensive than school-based surveys.

Personnel (64%) and transportation (18%) were the key cost drivers in Sudan, while personnel (38%) and survey consumables (27%) were the key drivers in South Sudan [8]. Whereas the survey in South Sudan was conducted only in a single state [32], the survey in Sudan was undertaken in all 18 states, which required an enormous workforce and a large amount of vehicles. Additionally, for the nationwide survey in Sudan, we incorporated federal-level supervision and an independent supervision mechanism for enhancing quality control, which led to a rise in costs. The participants of this study were 105,167 students from 1,772 schools in 390 ecological zones in 183 districts of all 18 states of Sudan. We suggest that the greatest share of costs for personnel and transportation should be reflected when budgeting future surveys for schistosomiasis and other intestinal helminthiases.

Apart from the key activities and costs, we provided considerable detail about the nationwide survey including the duration of the survey, the number of personnel by position and role, and the total number of each capital or recurrent item. We estimated the costs from the perspective of the donor and the Ministry of Health. However, if we had applied the societal perspective for costing [33, 34], the result would not have changed considerably because few items were not paid for in this survey. No volunteers were mobilized and parents or community members did not participate in this survey because it was conducted at the school level [35]. We used the same lifespan for capital items as in the reference case in the South Sudan [8]. The relatively short lifespan of capital items (i.e., 4 years for vehicles and 2 years for survey equipment) made the cost estimates more conservative.

The need for more sensitive diagnostics for intestinal helminths beyond the Kato-Katz technique, such as cathodic circulating antigen (CCA) testing or the ultrasensitive *Schistosoma* up-converting phosphor lateral flow circulating anodic antigen (UCP-LF CAA) assay, has received increasing attention in recent years [36–40]. A recent review suggested that the CCA assay is much more sensitive than the Kato-Katz assay in areas where the prevalence is lower than 50% [41]. We estimated the costs that would have been saved if we have applied the CCA tests for *S*. *mansoni* by referring to the previous studies, including a recent systematic review on CCA, in order to estimate the cost for the CCA test for *S*.*mansoni* [37–38,41]. We compared the results of this estimation with the actual costs incurred for our survey. For the unit cost of a urine-based point-of-contact CCA test alone, we used the lower and upper limits of US$ 1.46 and 1.76, respectively [41]. For labor costs of laboratory technicians, we referred to the previous study comparing the costs between the Kato-Katz and the CCA tests in Kenya [42]. In addition, we considered that we would need a smaller number of sample collectors or sampling days if we applied a CCA assay. We found that using the CCA assay would have reduced the survey costs by around US$ 70,314–99,318 (S8 Table). We recommend using the CCA assay for *S*. *mansoni* testing in areas where the prevalence is lower than 50%, including Sudan, in the next round of the survey.

Although over 1.5 billion people are thought to be affected by schistosomiasis and intestinal helminthiasis, the coverage of preventive chemotherapy is very low in many parts of the world, including Sudan [43]. It is critical to collect up-to-date information on the prevalence of schistosomiasis and intestinal helminthiases in order to better design national control programs, because doing so helps to invest limited resources more efficiently. In addition, presenting a nationwide mapping of prevalence could be an important tool to enhance national and global cooperation for controlling these diseases [44]. In recent years, the global NTD community has highlighted the importance of integrated NTD surveys and mapping because such approaches are more cost-effective for diseases with co-endemicity [32]. We expect the global health community to draw on this study when developing nationwide surveys of schistosomiasis and other intestinal helminthiases.

## Conclusion

Establishing a steering committee to develop and consensus a survey protocol, assessing the capabilities of potential staff (particularly laboratory technicians), and training laboratory technicians and data collectors are key components required to prepare a nationwide survey. Collecting lessons learned from frontline staff, sharing the results, and developing future plans with stakeholders are crucial activities after carrying out a nationwide survey. Key cost drivers are personnel and transportation. In particular, the personnel component accounts for a substantial share of the cost. The cost is expected to vary depending on the quantity and quality of existing laboratory facilities, equipment, and consumables, and the capability of laboratory technicians and sample collectors. Establishing central-level and independent supervision mechanisms to ensure the quality of the survey is equally important. It is worth noting that if a government finds a way to mobilize existing government officials with no additional payment using the health system already in place, the cost of a nationwide survey would be remarkably lower. In this survey, we sampled more students than the WHO recommended protocol because the mapping was done at smaller units than the district level to reflect the focalized nature of schistosomiasis infection. If the survey was done at the district level based on the WHO recommendation, the cost would have decreased by 51% mainly because of a smaller sample size. However, a substantial number of infected people would have been excluded from the MDA and a number of people negative for schistosomiasis would have received the MDA intervention unnecessarily, which would have led to lower cost-effectiveness compared with the revised methods in this survey. To the best of our knowledge, this is the first empirical study to investigate the key activities and cost drivers of a nationwide survey of schistosomiasis and other intestinal helminthiases.

## Supporting information

S1 Table Reference case scenario. (DOCX)

S2 Table Cost categories used and components included. (DOCX)

S3 Table Details on the workforce at the state level. (DOCX)

S4 Table Number of days for vehicles (rental car). (DOCX)

S5 Table Number of days for vehicles supported by the State Ministry of Health, Sudan. (DOCX)

S6 Table Number of days for other survey equipment/consumables. (DOCX)

S7 Table Details on the consumables by state. (DOCX)

S8 Table Difference in the costs between the Kato-Katz and the CCA tests. (DOCX)

S1 Dataset (SAV)
