# Supplementary material for: Cost and logistics implications of a nationwide survey of schistosomiasis and other intestinal helminthiases in Sudan: Key activities and cost components
Source: PLoS One. 2020 May 18;15(5):e0226586. doi: 10.1371/journal.pone.0226586 (PMC7233535; doi:10.1371/journal.pone.0226586)
Supplement: S1 Table — (DOCX) [file pone.0226586.s001.docx]

**S1 Table. Reference case scenario***

| **Parameter** | **Reference scenario** | **Explanation** |
| --- | --- | --- |
| Perspective | Provider | Use provider (KOICA/Ministry of Health, Sudan) perspective unless survey contributions were made by other parties and resources are available to measure all of these inputs, including the opportunity cost of survey participants. In this case present results from both the provider and societal viewpoints |
| Output | Ecological zone | Implementation unit (locality and ecological zone) surveyed to allow classification for intervention according to the threshold of Ministry of Health, Sudan. |
| Cost data | Include:  All survey costs (per-diem, transportation)  Time cost of head teachers, teachers  Donated items  Overhead costs | Use ingredient approach with cost categories as suggested in Supplementary Table 2. Quantities and prices need to be presented separately. Exclude research costs. Treat any equipment as a capital item if it is expected to last for more than one year and is purchased at a vale of US$ 100 or more. Calculate a daily financial cost for capital items by using straight line depreciation, and include the cost of each capital item for the number of survey days that it was used. |
| Currency | US$ | Costs should be presented in US$, indicating the year of conversion |
| Lifespan of capital items | Vehicles: 4 years | Establish average lifespan of capital items under local conditions and provide details on these in the narrative. Explore the impact of these assumptions in the sensitivity analysis. |
|  | Other equipment: 2 years |  |
| **Adjustment of financial costs to calculated economic costs** | |  |
| Annualisation | Lifespan | To obtain an equivalent annual cost for each capital outlay, an annualisation procedures needs to be followed. This requires an estimate of the lifespan of each capital item and a decision on the discount rate to be used. A proportion of the annaulised costs should be included as economic costs. We recommend that time (i.e. days of use of the equipment) is used for this calculation. |
| Discount rate | 3% | Base-case calculations should use 3%, to be consistent with World Bank recommendations. This should be varied in the sensitivity analysis (e.g. 1-10%). |
| **Reporting of results** | |  |
| Cost estimate | Cost per Implementation unit | Provide costs in US$  Specify year in which costs were calculated or adjusted to |

*We referred to the reference case scenario suggested by **Kolaczinski JH, et al.** [8]
