# Supplementary material for: Cost and logistics implications of a nationwide survey of schistosomiasis and other intestinal helminthiases in Sudan: Key activities and cost components
Source: PLoS One. 2020 May 18;15(5):e0226586. doi: 10.1371/journal.pone.0226586 (PMC7233535; doi:10.1371/journal.pone.0226586)
Supplement: S2 Table — (DOCX) [file pone.0226586.s002.docx]

**S2 Table. Cost categories used and components included***

| **Type/Category** | **Components** |
| --- | --- |
| **Capital costs** |  |
| Vehicles | Vehicles (excluding rental car)  Vehicle parts with a unit cost of USD 100 or more |
| Survey equipment | Microscope  Centrifuge  Refrigerator  Tablet PCs  Generator  Wifi Eggs |
| **Recurrent cots** |  |
| Vehicles | Rental car |
| Personnel | Daily allowances to federal level operational team, interviewers, sample collectors, laboratory technicians, laboratory assistants, cleaners, state coordinators, federal supervisors, independent supervisors, foreign experts, school teachers including headmasters |
|  |  |
| Survey consumables | Urine container, stool container, microscope slide tray, slide, cover, pincette, trans pippete, mess cylinder, wood stick, conical tube, tube rack, phenol, glycerol, malachite green oxalate, plastic box, mesh membrane, mask |
| Others | T-shirts (interviewers and sample collectors), Hat (interviewers and sample collectors), Gloves (interviewers and sample collectors), Supervision manual for federal-level supervisors Poster (bench aids for diagnosis of intestinal parasites of WHO), Questionnaire (printed version for contingency), Stationary (A4 sheets, pencils, notes for contingency), Delivery of survey consumables and equipment from Khartoum to each state, Accommodation for federal-level supervisors, Accommodation for foreign experts, Food items for foreign experts, Communication (air charge), Visas for foreign experts, Insurance for foreign experts, Air tickets for foreign experts, Software program for real-time data managing system using table PCs, Other miscellaneous petty cash (e.g. taxi fees, drinks for occasional meetings) |
|  |  |

*We referred to the cost categories and ingredients suggested by **Kolaczinski JH et al.** [ ]
