# Supplementary material for: Cost and logistics implications of a nationwide survey of schistosomiasis and other intestinal helminthiases in Sudan: Key activities and cost components
Source: PLoS One. 2020 May 18;15(5):e0226586. doi: 10.1371/journal.pone.0226586 (PMC7233535; doi:10.1371/journal.pone.0226586)
Supplement: S3 Table — (DOCX) [file pone.0226586.s003.docx]

**S3 Table. Details on the workforce at the state level**

| State | Days | State coordinator | Laboratory technician | Laboratory assistant | Cleaner | Interviewer,  Specimen collector |
| --- | --- | --- | --- | --- | --- | --- |
| Khartum | 24 | 1 | 14 | 5 | 1 | 12 |
| North Sudan | 20 | 1 | 12 | 4 | 1 | 12 |
| River Nile | 21 | 1 | 12 | 4 | 1 | 12 |
| Sennar | 16 | 1 | 12 | 4 | 1 | 12 |
| Blue Nile | 19 | 1 | 12 | 4 | 1 | 12 |
| Al gezira | 20 | 1 | 14 | 5 | 1 | 12 |
| North Kordofan | 22 | 1 | 12 | 4 | 1 | 12 |
| West Darfur | 27 | 1 | 12 | 4 | 1 | 12 |
| Center Darfur | 20 | 1 | 14 | 5 | 1 | 12 |
| East Darfur | 19 | 1 | 13 | 4 | 1 | 12 |
| White Nile | 27 | 1 | 14 | 5 | 1 | 12 |
| Red Sea | 22 | 1 | 12 | 4 | 1 | 12 |
| Kassala | 17 | 1 | 12 | 4 | 1 | 12 |
| Gadaref | 26 | 1 | 14 | 5 | 1 | 12 |
| West Kordofan | 32 | 1 | 18 | 6 | 1 | 16 |
| South Kordofan | 37 | 1 | 17 | 5 | 1 | 16 |
| North Darfur | 35 | 1 | 18 | 6 | 1 | 20 |
| South Darfur | 29 | 1 | 22 | 7 | 1 | 24 |
