# Supplementary material for: Cost and logistics implications of a nationwide survey of schistosomiasis and other intestinal helminthiases in Sudan: Key activities and cost components
Source: PLoS One. 2020 May 18;15(5):e0226586. doi: 10.1371/journal.pone.0226586 (PMC7233535; doi:10.1371/journal.pone.0226586)
Supplement: S4 Table — (DOCX) [file pone.0226586.s004.docx]

**S4 Table. Number of days for vehicles (rental car)**

| State | | No | days | total days |
| --- | --- | --- | --- | --- |
|  |  |  |  |  |
| other areas | Khartum | 5 | 24 | 120 |
|  | North Sudan | 4 | 23 | 92 |
|  | River Nile | 3 | 21 | 63 |
|  | Sennar | 4 | 22 | 88 |
|  | Blue Nile | 4 | 19 | 76 |
|  | Al gezira | 5 | 29 | 145 |
|  | North Kordofan | 5 | 27 | 135 |
|  | White Nile | 5 | 27 | 135 |
|  | Red Sea | 3 | 24 | 72 |
|  | Kassala | 3 | 24 | 72 |
|  | Gadaref | 6 | 27 | 162 |
|  | West Kordofan | 6 | 38 | 228 |
|  | South Kordofan | 6 | 38 | 228 |
| Darfur areas | South Darfur | 6 | 38 | 214 |
|  | East Darfur | 7 | 29 | 206 |
|  | North Darfur | 8 | 34 | 274 |
|  | Central Darfur | 3 | 31 | 79 |
