# Supplementary material for: Cost and logistics implications of a nationwide survey of schistosomiasis and other intestinal helminthiases in Sudan: Key activities and cost components
Source: PLoS One. 2020 May 18;15(5):e0226586. doi: 10.1371/journal.pone.0226586 (PMC7233535; doi:10.1371/journal.pone.0226586)
Supplement: S5 Table — (DOCX) [file pone.0226586.s005.docx]

**S5 Table. Number of days for vehicles supported by the State Ministry of Health, Sudan**

| State | No | Days | Total days |
| --- | --- | --- | --- |
| Khartum | 1 | 24 | 24 |
| North Sudan | 1 | 20 | 20 |
| River Nile | 1 | 21 | 21 |
| Sennar | 1 | 16 | 16 |
| Blue Nile | 1 | 19 | 19 |
| Al gezira | 1 | 20 | 20 |
| North Kordofan | 1 | 22 | 22 |
| West Darfur | 1 | 27 | 27 |
| Center Darfur | 0 | 20 | 0 |
| East Darfur | 0 | 19 | 0 |
| White Nile | 0 | 27 | 0 |
| Red Sea | 1 | 22 | 22 |
| Kassala | 1 | 17 | 17 |
| Gadaref | 2 | 26 | 52 |
| West Kordofan | 1 | 32 | 32 |
| South Kordofan | 1 | 37 | 37 |
| North Darfur | 1 | 35 | 35 |
| South Darfur | 2 | 29 | 58 |
