# Supplementary material for: Cost and logistics implications of a nationwide survey of schistosomiasis and other intestinal helminthiases in Sudan: Key activities and cost components
Source: PLoS One. 2020 May 18;15(5):e0226586. doi: 10.1371/journal.pone.0226586 (PMC7233535; doi:10.1371/journal.pone.0226586)
Supplement: S6 Table — (DOCX) [file pone.0226586.s006.docx]

**S6 Table. Number of days for other survey equipment/consumables**

| State | Days |
| --- | --- |
| Khartum | 24 |
| North Sudan | 20 |
| River Nile | 21 |
| Sennar | 16 |
| Blue Nile | 19 |
| Al gezira | 20 |
| North Kordofan | 22 |
| West Darfur | 27 |
| Center Darfur | 20 |
| East Darfur | 19 |
| White Nile | 27 |
| Red Sea | 22 |
| Kassala | 17 |
| Gadaref | 26 |
| West Kordofan | 32 |
| South Kordofan | 37 |
| North Darfur | 35 |
| South Darfur | 29 |
