# Supplementary material for: Cost and logistics implications of a nationwide survey of schistosomiasis and other intestinal helminthiases in Sudan: Key activities and cost components
Source: PLoS One. 2020 May 18;15(5):e0226586. doi: 10.1371/journal.pone.0226586 (PMC7233535; doi:10.1371/journal.pone.0226586)
Supplement: S7 Table — (DOCX) [file pone.0226586.s007.docx]

**S7 Table. Details on the consumables by state**

| State | Microscope | Centrifuge | Refrigerator | Urine container | Stool container | Slide | Cover | Pipet | Conical tube | Microscope slide tray | Mess cylinder | Pincette | Mesh membrane | Mask | Gloves |
| --- | --- | --- | --- | --- | --- | --- | --- | --- | --- | --- | --- | --- | --- | --- | --- |
| Khartum | 1 | 1 | 1 | 10,000 | 10,000 | 60,000 | 10,000 | 24,000 | 150 | 15 | 1 | 3 | 5 | 3 | 5 |
| North Sudan | 2 | 1 | 1 | 7,500 | 7,500 | 25,000 | 8,000 | 20,000 | 50 | 15 | 1 | 3 | 3 | 2 | 5 |
| River Nile | 1 | 1 | 1 | 7,000 | 7,000 | 25,000 | 7,500 | 20,000 | 50 | 15 | 1 | 3 | 3 | 2 | 5 |
| Sennar | 1 | 1 | 1 | 6,500 | 6,500 | 20,000 | 8,000 | 20,000 | 50 | 15 | 1 | 3 | 3 | 2 | 5 |
| Blue Nile | 2 | 2 | 1 | 8,500 | 8,500 | 28,000 | 8,500 | 21,000 | 50 | 15 | 1 | 3 | 3 | 2 | 5 |
| Al gezira | 1 | 1 | 1 | 12,000 | 12,000 | 30,000 | 12,000 | 28,000 | 150 | 15 | 1 | 3 | 4 | 4 | 5 |
| North Kordofan | 3 | 2 | 1 | 8,500 | 8,500 | 25,000 | 8,500 | 20,000 | 50 | 15 | 1 | 3 | 4 | 2 | 5 |
| West Darfur | 3 | 2 | 1 | 9,500 | 9,500 | 25,000 | 9,000 | 21,000 | 50 | 15 | 1 | 3 | 4 | 2 | 5 |
| Center Darfur | 3 | 3 | 1 | 11,000 | 11,000 | 30,000 | 12,000 | 28,000 | 50 | 20 | 1 | 3 | 6 | 4 | 5 |
| East Darfur | 3 | 3 | 1 | 9,500 | 9,500 | 25,000 | 9,500 | 21,000 | 50 | 20 | 1 | 3 | 6 | 3 | 5 |
| White Nile | 1 | 3 |  | 14,000 | 14,000 | 35,000 | 14,000 | 30,000 | 200 | 20 | 1 | 3 | 7 | 4 | 5 |
| Red Sea | 1 | 5 | 1 | 6,500 | 6,500 | 22,000 | 7,500 | 19,000 | 50 | 20 | 1 | 3 | 6 | 3 | 5 |
| Kassala | 3 | 5 | 1 | 7,000 | 7,000 | 20,000 | 8,500 | 19,000 | 50 | 25 | 1 | 3 | 8 | 3 | 5 |
| Gadaref | 3 | 5 | 1 | 12,000 | 12,000 | 30,000 | 8,500 | 28,000 | 150 | 25 | 1 | 3 | 8 | 3 | 5 |
| West Kordofan | 3 | 5 | 1 | 20,000 | 20,000 | 65,000 | 18,500 | 50,000 | 250 | 35 | 1 | 3 | 7 | 4 | 5 |
| South Kordofan | 3 | 5 | 1 | 14,500 | 14,500 | 35,000 | 13,500 | 36,000 | 200 | 35 | 1 | 3 | 7 | 3 | 5 |
| North Darfur | 3 | 6 | 1 | 13,000 | 13,000 | 30,000 | 11,500 | 35,000 | 200 | 40 | 1 | 3 | 7 | 4 | 5 |
| South Darfur | 3 | 6 | 1 | 23,000 | 23,000 | 70,000 | 25,000 | 60,000 | 200 | 40 | 1 | 3 | 9 | 4 | 5 |
| Total | 40 | 57 | 17 | 200000 | 200000 | 600000 | 200000 | 500000 | 2000 | 400 | 18 | 54 | 100 | 54 | 90 |
