# Supplementary material for: Cost and logistics implications of a nationwide survey of schistosomiasis and other intestinal helminthiases in Sudan: Key activities and cost components
Source: PLoS One. 2020 May 18;15(5):e0226586. doi: 10.1371/journal.pone.0226586 (PMC7233535; doi:10.1371/journal.pone.0226586)
Supplement: S8 Table — (DOCX) [file pone.0226586.s008.docx]

**S8 Table. Difference in the costs between the Kato-Katz and the CCA tests**

|  | No. of lab. technician | No. of total days | No of days for stool exam. | Payment | No. of Interviewer/  sample collector | No. of stool collectors | No of days | Payment | No. of students | KK kits  Unit price | Times of exam  /person | Sum | SUM |
| --- | --- | --- | --- | --- | --- | --- | --- | --- | --- | --- | --- | --- | --- |
| Khartum | 14 | 24 | 12 | 8975 | 12 | 3 | 24 | 3847 | 5514 | 0.3 | 2 | 3308 |  |
| North Sudan | 12 | 23 | 12 | 7373 | 12 | 3 | 23 | 3686 | 3578 | 0.3 | 2 | 2147 |  |
| River Nile | 12 | 21 | 11 | 6731 | 12 | 3 | 21 | 3366 | 2988 | 0.3 | 2 | 1793 |  |
| Sennar | 12 | 22 | 11 | 7052 | 12 | 3 | 22 | 3526 | 2763 | 0.3 | 2 | 1658 |  |
| Blue Nile | 12 | 19 | 10 | 6090 | 12 | 3 | 19 | 3045 | 3709 | 0.3 | 2 | 2225 |  |
| Al gezira | 14 | 29 | 15 | 10845 | 12 | 3 | 29 | 4648 | 5710 | 0.3 | 2 | 3426 |  |
| North Kordofan | 12 | 27 | 14 | 8655 | 12 | 3 | 27 | 4327 | 3578 | 0.3 | 2 | 2147 |  |
| West Darfur | 12 | 27 | 14 | 8655 | 12 | 3 | 27 | 4327 | 3944 | 0.3 | 2 | 2366 |  |
| Center Darfur | 14 | 31 | 16 | 11593 | 12 | 3 | 31 | 4968 | 3175 | 0.3 | 2 | 1905 |  |
| East Darfur | 13 | 29 | 15 | 10070 | 12 | 3 | 29 | 4648 | 4252 | 0.3 | 2 | 2551 |  |
| White Nile | 14 | 27 | 14 | 10097 | 12 | 3 | 27 | 4327 | 6579 | 0.3 | 2 | 3947 |  |
| Red Sea | 12 | 24 | 12 | 7693 | 12 | 3 | 24 | 3847 | 2425 | 0.3 | 2 | 1455 |  |
| Kassala | 12 | 24 | 12 | 7693 | 12 | 3 | 24 | 3847 | 3441 | 0.3 | 2 | 2065 |  |
| Gadaref | 14 | 27 | 14 | 10097 | 12 | 3 | 27 | 4327 | 5821 | 0.3 | 2 | 3493 |  |
| West Kordofan | 18 | 38 | 19 | 18271 | 16 | 4 | 38 | 8120 | 10190 | 0.3 | 2 | 6114 |  |
| South Kordofan | 17 | 38 | 19 | 17256 | 16 | 4 | 38 | 8120 | 8402 | 0.3 | 2 | 5041 |  |
| North Darfur | 18 | 34 | 17 | 16348 | 20 | 5 | 34 | 9082 | 6562 | 0.3 | 2 | 3937 |  |
| South Darfur | 22 | 38 | 19 | 22331 | 24 | 6 | 38 | 12181 | 14048 | 0.3 | 2 | 8429 |  |
|  |  |  |  | 195826 |  |  |  | 94240 | 96679 | 0.3 | 2 | 58007 | 348073 |

**S8 Table. Difference in the costs between the Kato-Katz and the CCA tests** (continued)

| CCA kits | Days for exam | Payment | No. of students | Lower | Upper | Sum (lower) | Sum (upper) | Difference between CCA and KK test (upper) | Difference  between CCA and KK test (lower) |
| --- | --- | --- | --- | --- | --- | --- | --- | --- | --- |
| 14 | 8 | 6137 | 5514 | 8050 | 9705 |  |  |  |  |
| 12 | 6 | 3982 | 3578 | 5224 | 6297 |  |  |  |  |
| 12 | 5 | 3326 | 2988 | 4362 | 5259 |  |  |  |  |
| 12 | 5 | 3075 | 2763 | 4034 | 4863 |  |  |  |  |
| 12 | 6 | 4128 | 3709 | 5415 | 6528 |  |  |  |  |
| 14 | 8 | 6355 | 5710 | 8337 | 10050 |  |  |  |  |
| 12 | 6 | 3982 | 3578 | 5224 | 6297 |  |  |  |  |
| 12 | 7 | 4390 | 3944 | 5758 | 6941 |  |  |  |  |
| 14 | 5 | 3534 | 3175 | 4636 | 5588 |  |  |  |  |
| 13 | 7 | 4732 | 4252 | 6208 | 7484 |  |  |  |  |
| 14 | 10 | 7322 | 6579 | 9605 | 11579 |  |  |  |  |
| 12 | 4 | 2699 | 2425 | 3541 | 4268 |  |  |  |  |
| 12 | 6 | 3830 | 3441 | 5024 | 6056 |  |  |  |  |
| 14 | 9 | 6479 | 5821 | 8499 | 10245 |  |  |  |  |
| 18 | 12 | 11341 | 10190 | 14877 | 17934 |  |  |  |  |
| 17 | 10 | 9351 | 8402 | 12267 | 14788 |  |  |  |  |
| 18 | 8 | 7304 | 6562 | 9581 | 11549 |  |  |  |  |
| 22 | 13 | 15635 | 14048 | 20510 | 24724 |  |  |  |  |
|  |  | 107604 |  | 141151 | 170155 | 248755 | 277759 | 99318 | 70314 |
